# Genomic identification and characterization of the pseudoautosomal region in highly differentiated avian sex chromosomes

**DOI:** 10.1038/ncomms6448

**Published:** 2014-11-07

**Authors:** Linnéa Smeds, Takeshi Kawakami, Reto Burri, Paulina Bolivar, Arild Husby, Anna Qvarnström, Severin Uebbing, Hans Ellegren

**Affiliations:** 1 Department of Evolutionary Biology, Evolutionary Biology Centre, Uppsala University, Norbyvägen 18D, SE-752 36 Uppsala, Sweden; 2 Department of Animal Ecology, Evolutionary Biology Centre, Uppsala University, Norbyvägen 18D, SE-752 36 Uppsala, Sweden; 3 Department of Biology, Norwegian University of Science and Technology, N-7491 Trondheim, Norway

## Abstract

The molecular characteristics of the pseudoautosomal region (PAR) of sex chromosomes remain elusive. Despite significant genome-sequencing efforts, the PAR of highly differentiated avian sex chromosomes remains to be identified. Here we use linkage analysis together with whole-genome re-sequencing to uncover the 630-kb PAR of an ecological model species, the collared flycatcher. The PAR contains 22 protein-coding genes and is GC rich. The genetic length is 64 cM in female meiosis, consistent with an obligate crossing-over event. Recombination is concentrated to a hotspot region, with an extreme rate of >700 cM/Mb in a 67-kb segment. We find no signatures of sexual antagonism and propose that sexual antagonism may have limited influence on PAR sequences when sex chromosomes are nearly fully differentiated and when a recombination hotspot region is located close to the PAR boundary. Our results demonstrate that a very small PAR suffices to ensure homologous recombination and proper segregation of sex chromosomes during meiosis.

## Abstract (web summary)

The genetic basis of sex chromosome pseudoautosomal regions (PAR) in organisms with female heterogamety is largely unknown. Smeds *et al.* provide the first molecular characterization of the PAR in birds with differentiated sex chromosomes and show a potential recombination hotspot and no evidence for strong sexual antagonism in this region.

## Graphical abstract

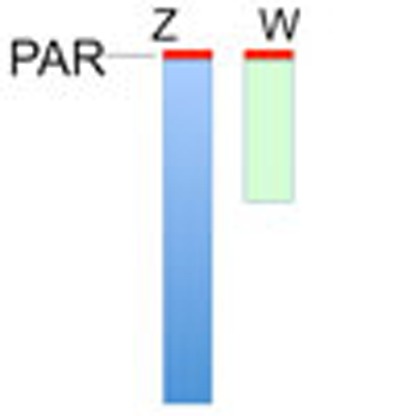

Cessation of recombination between diverging sex chromosomes makes the non-recombining sex chromosome vulnerable to a number of degenerative forces. Inevitable accumulations of deleterious mutations through the process of Muller’s ratchet, selective sweeps and reduced effective population size are examples of such forces that act to increase the mutation load of the sex-limited chromosome, that is, the Y chromosome in male heterogametic organisms and the W chromosome in female heterogametic organisms^1^,^2^,^3^. Gene sequence and activity for most loci will therefore deteriorate unless they are under strong selection in the heterogametic sex^4^,^5^. This process will continue as recombination cessation spreads along the sex chromosomes in the heterogametic sex^6^.

However, the need for chromosome pairing and an obligate crossing-over event to ensure proper chromosomal segregation at meiosis is thought to, in most cases, strongly select against complete loss of sex chromosome recombination^7^,^8^,^9^; but see ref. 10. For this to be possible, it is necessary that the differentiated sex chromosomes maintain sequence homology in at least one common region shared between X and Y, or Z and W, where homologous recombination can take place^11^,^12^. This is referred to as the pseudoautosomal region (PAR)^9^ and cytology has since long established that meiotic pairing and crossing-over between differentiated sex chromosomes of diverse organism groups are concentrated to the PAR^13^.

At the onset of sex chromosome differentiation, which may be triggered by selection against recombination around a sex-determining locus^5^, the proto-sex chromosomes correspond to an ordinary pair of autosomes. However, as soon as recombination restriction is established in at least a small region of this pair, the distinction between the non-recombining region and the pseudoautosomal part of the sex chromosomes becomes apparent. In newly evolved (young) sex chromosome systems^14^, the PAR may constitute a major part of the X (Z) chromosome. However, the PAR shrinks as recombination suppression spreads. PAR is thus a dynamic entity and additions, losses or transpositions of chromosomal segments to sex chromosomes may add to the dynamics of the evolution of PARs^15^.

It is fair to say that the PAR represents one of the least well-characterized parts of the genome. Genomic data on the character and structure of the PAR in old and differentiated sex chromosomes mainly come from humans and other mammals^16^,^17^. Birds have come to constitute the most well-studied group of organisms in terms of sex chromosome evolution under female heterogamety^18^,^19^. Chicken *Gallus gallus* is a main avian model, and a draft genome sequence was presented already 10 years ago^20^. However, despite a number of studies investigating how the chicken Z and W chromosomes became differentiated^21^,^22^,^23^,^24^, the PAR still remains to be molecularly identified and this is also the case for all other birds with well-differentiated sex chromosomes^25^ (in the most basal lineage of contemporary birds, Paleognathae (ostrich and allies, representing <1% of avian species), sex chromosomes have remained largely undifferentiated^26^,^27^; Supplementary Note 1). This has led to the idea that the avian PAR might in most cases be very small or shows some peculiar molecular features that hinder its identification.

Here we describe the identification of an avian PAR using a combination of high-density genetic linkage analysis and whole-genome re-sequencing in the collared flycatcher *Ficedula albicollis*. We find that the PAR is a 630-kb region in one of the ends of the Z chromosome and by performing population genomic and molecular evolutionary analyses, we test theoretical predications^9^,^28^,^29^,^30^ for the genetics and evolution of pseudoautosomal sequences.

## Results

### Identification of the PAR based on linkage analysis

By selecting markers from essentially all scaffolds in a draft assembly of the collared flycatcher genome^31^, we recently developed a custom single-nucleotide polymorphism (SNP) array and obtained a genome-wide high-density genetic linkage map (3,249 cM) by genotyping large multi-generational pedigrees from a natural population^32^,^33^. This included a 161-cM male map of the Z chromosome, which showed no female recombination and testifies of advanced sex chromosome differentiation in this species. To identify the PAR, we focused on SNPs heterozygous in both males and females in a pool of unmapped markers that were not linked to any of the 33 autosomal linkage groups^33^. Seven of these previously unlinked markers from three small scaffolds (N00298, three markers in 436.0 kb; N00378, three markers in 182.2 kb; N02597, one marker in 2.3 kb) showed highly significant two-point linkage in both male and female meioses to several markers located close to one end of the Z chromosome linkage map and to each other (Supplementary Table 1; Supplementary Fig. 1).

**Figure 1:**
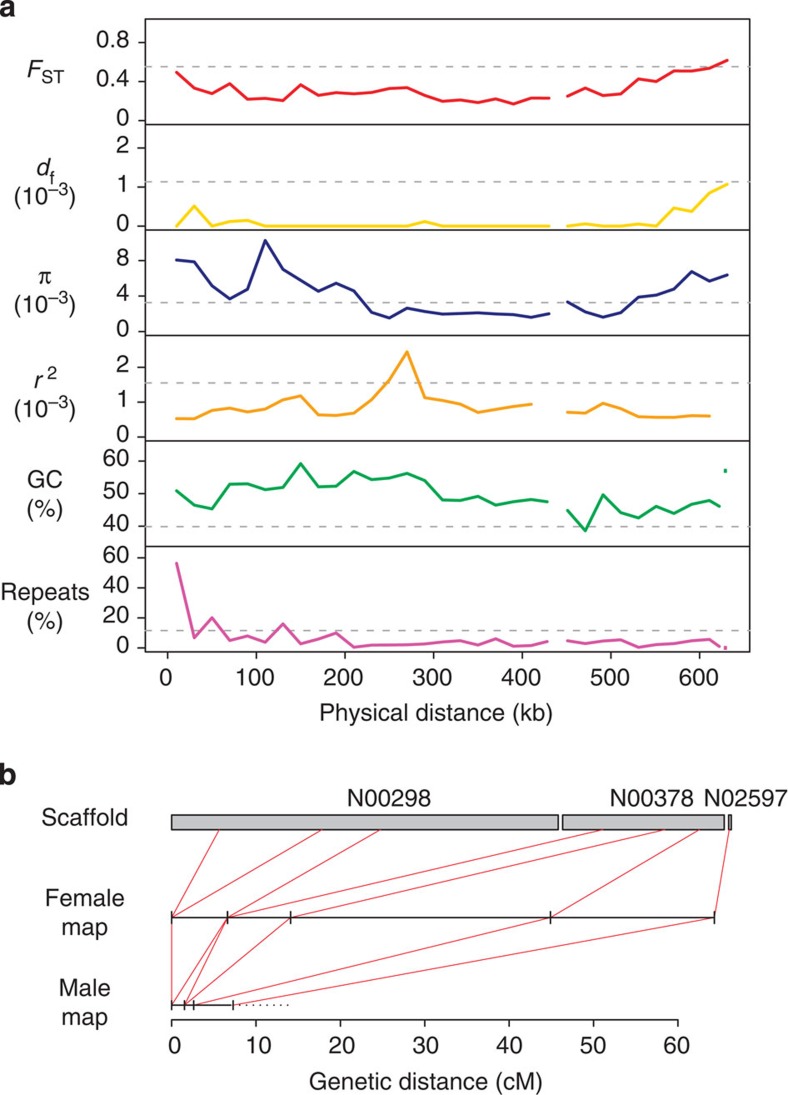
Characteristics of the flycatcher PAR. (**a**) Estimates of population genetic and genomic parameters in 20 kb windows across the PAR in comparison with the mean for the rest of the Z chromosomes (dashed line in each panel). From top to bottom: between-species differentiation *F*_ST_, density of fixed differences between species *d*_f_, nucleotide diversity (*π*), LD (*r*^*2*^), GC content (%) and repeat density (%). (**b**) Physical and genetic description of the PAR showing the three scaffolds assigned to PAR and genetic maps for females and males, respectively, in this region.

We built a new map of the Z chromosome that with strong support placed markers from N00298, N00378 and N02597 distal to all other markers on the chromosome (Table 1) and extended the Z chromosome linkage map as measured in male meiosis with 7.3 cM and the Z chromosome assembly with 630 kb (Fig. 1b; Supplementary Note 2). There was a dramatic sex difference in the amount of recombination in this region with a female map length of 64.3 cM (Fig. 1b), corresponding to a female recombination rate of 102.1 cM/Mb. With a genetic distance >50 cM, the data are compatible with an obligate crossing-over in female meiosis, consistent with expectations for a PAR. Moreover, female recombination was not uniformly distributed across the 630 kb but was concentrated to an ~150-kb hotspot region (with an extreme rate of 747 cM/Mb in the 67-kb interval between markers N00378:115359 and N02597:626) distal to the boundary with the rest of the Z chromosome.

### Identification of the PAR based on read depth

**Figure 2:**
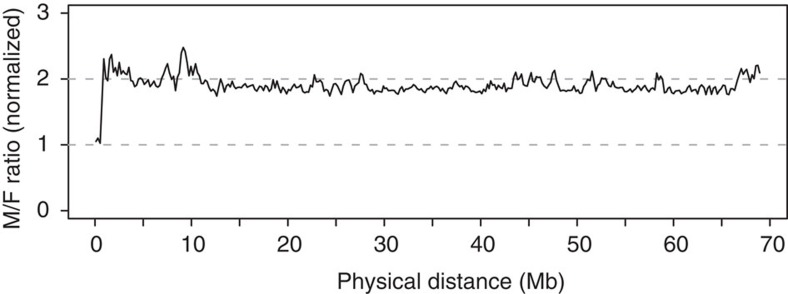
Sex-specific gene expression. Male-to-female (M/F) coverage ratio for 200 kb windows along the Z chromosome. Coverage was normalized by the average M/F ratio of autosomal scaffolds.

An independent way to identify a PAR is to contrast depth of coverage in re-sequencing of males and females^26^,^34^,^35^. Specifically, while autosomes and PARs should show similar coverage in male and female sequencing, the region of the Z chromosome that does not recombine with the W chromosome in female meiosis should show twofold higher coverage in males. We therefore performed whole-genome re-sequencing of population samples of males and females and mapped reads to the assembly. This clearly demonstrated a twofold higher male coverage across the Z chromosome, with the exception of scaffolds N00298, N00378 and N02597, where males and females had equal coverage (Fig. 2; Supplementary Fig. 2). On the basis of the described evidence, we now define these three scaffolds as the collared flycatcher PAR, the first detected PAR in a pair of well-differentiated avian sex chromosomes (Supplementary Note 1). The physical length of the PAR corresponds to ≤1% of the total length of the Z chromosome and implies that cessation of female recombination has spread over ≥99% of the sex chromosomes. Given previous failure to identify the PAR in neognath birds (all birds but ratites and tinamous), a small PAR may be a common feature of avian sex chromosomes (Supplementary Note 3). Moreover, this resembles the situation for a recently identified small PAR in a female heterogametic flatfish^36^. We acknowledge that since there are gaps between scaffolds within the PAR, as well as between PAR and the rest of the Z chromosome, the complete PAR sequence and the precise PAR boundary remain to be determined. The same applies for any telomeric sequence.

### Genomic characteristics of the PAR and PAR genes

The flycatcher PAR contains 16 known and 6 *de novo*-predicted protein-coding genes (Supplementary Table 2). This implies a higher gene density in the PAR than in the Z chromosome overall, both expressed as the number of genes per Mb (34.9 versus 10.0) and the amount of coding sequence per base pair (bp; 0.048 versus 0.016, non-parametric boostrap re-sampling, *P*<10^−5^). Consistent with a tight organization, repeat content was lower in the PAR (0.064 per bp) than in the rest of the Z chromosomes (0.116, *P*=0.081; Fig. 1a; Table 2). The high rate of recombination in the PAR may have generated an excess of deletion mutations^37^ and may also have increased the efficiency of selection against deleterious insertions of repetitive elements. A high rate of recombination might also be expected to have left a footprint on the base composition of PAR^38^ via GC-biased gene conversion^39^,^40^,^41^. Indeed, the mean GC content (49.2%) was significantly higher than in the rest of the Z chromosomes (mean=39.9%, range=36.4–48.3%, *P*=0.00056; Fig. 1a; Table 2).

Recombination rate may affect the rate of sequence evolution in different ways. We made three-species alignments of coding sequences of flycatcher, zebra finch (*Taeniopygia guttata*) and chicken to estimate branch-specific substitution rates in the flycatcher lineage (Table 2). The mean synonymous-to-non-synonymous substitution rate ratio (*d*_N_/*d*_S_) of PAR genes was lower than that of other Z-linked genes (0.095 versus 0.171, *P*=0.030, Wilcoxon test), consistent with more efficient removal of slightly deleterious mutations in the PAR due to reduced Hill–Robertson interference. More surprisingly, *d*_S_ of PAR genes (0.130) was significantly higher than that of other Z-linked genes (0.078, *P*=0.0014). This cannot be explained by constraints at synonymous sites^42^ or male-biased mutation^43^ because both would act in the opposite direction, with a higher substitution rate on the rest of the Z chromosomes. Timing and mechanisms of recombination and the formation of double-strand breaks in female germ line have been shown to differ between PAR and autosomes in chicken^44^, and Z–W pairing is error prone^45^. This might translate into a situation where the extraordinary high rate of recombination implies an increased rate of mutation in the PAR.

### PAR and sexual antagonism

Because PAR sequences may be polymorphic in both sexes but yet show an association with sex, increasingly so closer to the boundary with the sex-determining region^29^, the stage is potentially set for a strong role of sexual antagonism^46^ on the character and evolution of genetic diversity in PAR^9^. Recently, several evolutionary genetic predictions pertinent to pseudoautosomal sequences have been developed^9^,^28^,^29^,^30^,^47^. For example, since sexual antagonism can favour the maintenance of polymorphisms by selection for alternate alleles in males and females, genetic diversity in PARs should be high^30^. Moreover, the rather unusual scenario of allele frequency differences between males and females may apply^48^, due to the formation of linkage disequilibria between sexually antagonistic alleles and the Z chromosome or the W chromosome^28^. To test this, we used whole-genome re-sequencing of 10 males and 10 females to assess levels of noncoding nucleotide diversity (*π*). We found that diversity in the PAR (mean *π*=0.0034) was not significantly different from the rest of the Z chromosome (mean *π*=0.0032; non-parametric bootstrap re-sampling, *P*=1; Fig. 1a; Table 2). Moreover, there was no detectable differentiation between males and females in the PAR (*F*_ST_=0.007±0.011 s.d.) or in the rest of the Z chromosome (0.012±0.023 s.d.), as would have been the case with sex differences in allele frequencies. Females were heterozygous throughout the PAR at a rate identical to that in males.

In none of these cases were there any deviating signals close to the boundary with the sex determining region. Levels of linkage disequilibrium (LD) in the PAR were lower (mean *r*^2^=0.00087) than in the rest of the Z chromosomes (mean *r*^2^=0.00157, Wilcoxon test, *P*=3.1e^−10^; Fig. 1a), with a mean distance of LD decaying to *r*^2^=0.1 of 45 bp in the PAR and of 1,558 bp in the rest of the Z chromosomes (Supplementary Fig. 3). As a side note, genomic differentiation in comparison with the closely related pied flycatcher (*F. hypoleuca*) was much lower in PAR than in the rest of the Z chromosome (*F*_ST_: 0.372 versus 0.555, *P*=0.00051; *d*_f_: 0.0001 versus 0.0011, *P*=0.00051). This provides support for an increased rate of sex-linked lineage sorting. Enhanced differentiation of sex chromosomes observed in this^31^ and other speciation models^49^ can thereby be explained by the lower effective population size of sex chromosomes compared with autosomes and PARs.

If sexual antagonism is prevalent, theory predicts an over-representation of genes with sex-specific functions on the sex chromosomes^50^. However, none of the annotated PAR genes (Supplementary Table 2) had known function in male or female reproduction. Another prediction is that sex-specific expression, or sex-biased gene expression as a means to resolve sexual conflict^50^,^51^, should be evident. We analysed expression profiles using RNA-sequencing from seven non-reproductive tissues, plus testis and ovary, for five males and five females. Twenty PAR genes were expressed in at least one of the tissues analysed and expression breadth did not deviate from other Z-linked genes (mean *τ* of 0.601 and 0.657, respectively, *P*=0.239). One PAR gene (ENSFALG00000011567, predicted transcript) showed testis-specific expression while none showed ovary-specific expression, which is at a level expected by chance given the overall frequency of testis- and ovary-specific genes in the genome (probability of 0.135). The tissue-averaged male-to-female expression ratio for PAR genes varied between 0.76 and 1.20, with a mean of 0.95 (similar to the autosomal average, 1.02). This made a marked contrast to the situation for other genes on the flycatcher Z chromosome, which had a mean male-to-female ratio of 1.40 (*P*<1e−10). There is ample evidence for pervasive male-biased gene expression (incomplete dosage compensation) in the Z chromosome in this^52^ and other avian species^53^,^54^. In summary, annotation and expression of genes in the PAR provide no strong indication of sexual antagonism.

**Figure 3:**
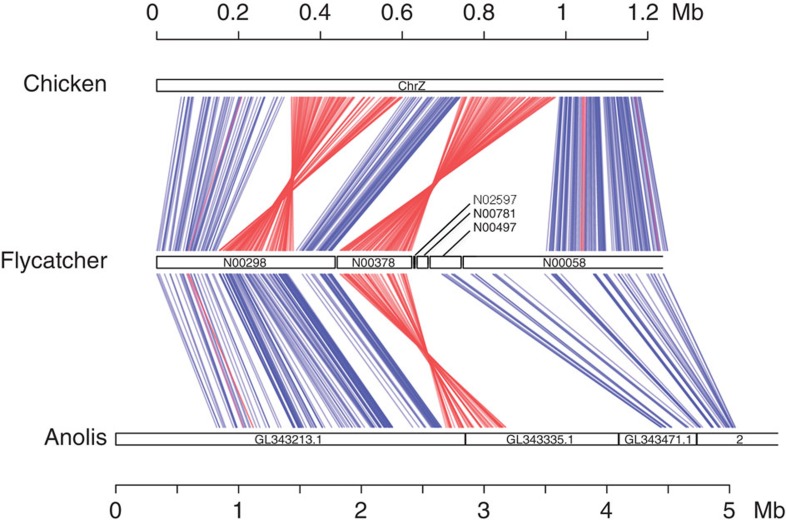
Comparative genome organization of flycatcher PAR. Homologous sequences of chicken, flycatcher and *Anolis* lizard including the flycatcher PAR region and the distal 600 kb of the non-recombining part of the flycatcher Z chromosome. Each line joins homologous regions identified as anchors by the program LASTZ. Blue lines represent identical orientation of homologous sequences, red lines represent inverted orientation. Scaffolds N02597, N00781 and N00497 contain no genes, meaning that establishment of homology is difficult. Note the difference in scale between chicken and flycatcher (upper scale) and lizard (lower scale).

One possible explanation for the failure to verify theoretical expectations based on sexual antagonism in the evolution of flycatcher PAR sequences includes frequent turnover of the PAR by interchromosomal rearrangements. However, this explanation is highly unlikely because of a high degree of conservation of this region in birds. Genomic alignment of flycatcher and chicken revealed that the flycatcher PAR corresponds to one of the terminal regions also of the chicken Z chromosome (Fig. 3), with completely conserved gene content. Two inversions distinguish gene order between the flycatcher PAR and the homologous region of the chicken Z chromosome (Fig. 3). Using *Anolis* lizard as an outgroup suggests that the most distal inversion arose in the lineage leading to chicken, subsequent to the split of the chicken and flycatcher lineages 80 myr ago^55^. The other discrepancy in gene order between chicken and flycatcher coincides exactly with scaffold N00378. This scaffold was oriented with a logarith of the odds (LOD) score support >3 in the flycatcher linkage map and orientation was also supported by mate-pair data (Supplementary Note 1). Our data therefore show that, despite some internal inversions, the sequence content of the flycatcher PAR has remained stable during avian evolution. In general, the avian karyotype is extremely conserved with very few interchromosomal rearrangements^56^; in fact, flycatcher and zebra finch chromosomes are completely syntenic without fusions, fissions or translocations detectable with the resolution given standard methodology^33^.

## Discussion

Identification of the collared flycatcher PAR was achieved by indisputable support for genetic linkage of markers from three previously unassigned scaffolds to the Z chromosome, equal depth of coverage in male and female genomic re-sequencing, evidence for an obligate crossing-over in female meiosis and presence of heterozygote sites across this region in females. To our knowledge, this represents the first identification and extensive sequencing and genetic analysis of a PAR in a pair of highly differentiated avian sex chromosomes (Supplementary Note 1). It includes estimation of the size, boundary, sequence and gene content of the PAR, and analyses of gene expression and several population genetic and molecular evolutionary parameters. We find that the PAR is intermediate to autosomal and sex-linked sequences in several evolutionary and genomic respects. It is interesting to note that the recent identification of the first PAR in a female heterogametic fish revealed a very similar size, number of genes, repeat content and male:female expression ratio as for the PAR in flycatcher^36^.

There has been considerable recent interest in the evolutionary expectations for pseudoautosomal sequences, based on sexual antagonism^9^,^28^,^29^,^30^,^47^. Much of this theoretical work remains to be empirically tested and our data provide one of the first opportunities to do so with a population genomic approach. This is particularly the case when it comes to female heterogametic sex chromosomes. However, we found no evidence for a role of sexual antagonism on sequence content or evolution. It is possible that theoretical predictions for the evolution of PAR sequences are not applicable to a situation of highly differentiated sex chromosomes, as observed in flycatchers. First, with most of the observed recombination concentrated close to the PAR boundary, distal PAR sequences will be effectively autosomal. However, as recombination hotspots may be ephemeral^57^, this pattern may have changed over time. Second, there might be constraints to sexual antagonism in a small PAR that is defined by the particular set of a limited number of genes that happen to reside in the terminal part of the Z chromosome. This situation may have been different at earlier stages of sex chromosome evolution. A widely accepted model of sex chromosome evolution implies gradual or sequential expansion of recombination restriction between the Z (or X) and W (or Y) chromosomes, and the concomitant contraction of the PAR, driven by selection for linkage between sexually antagonistic alleles and the sex-determining region^58^. After recombination restriction, such loci will subsume into the non-recombining region to become truly sex limited, thereby reducing signals of antagonism in the contracted PAR. An extension of this hypothesis is a negative feedback loop in which the impetus for further expansion of the non-recombining region of sex chromosomes is increasingly reduced with a decreasing number of potential targets for sexual antagonism in the remaining PAR.

## Methods

### Identification of the PAR based on linkage analysis

We used a natural population of collared flycatchers breeding on the Baltic Sea island Öland (sampling conducted according to permissions and rules of the Swedish ethics committee for wild animals) and a custom 50K SNP array^32^ to obtain genotypes of 655 individuals from four-generation pedigrees for linkage analysis^33^. Genotyping was performed with an Illumina iScan instrument. The array had purposedly been designed to include highly variable SNPs from essentially all scaffolds >25 kb in a preliminary assembly version of the flycatcher genome^32^. After filtering for deviations from Hardy–Weinberg equilibrium and Mendelian inheritance, linkage analysis was performed using CRI-MAP 2.503 (ref. 59) developed by Ian Evans and Jill Maddox. Genotype data were initially used to construct a high-density linkage map, comprising 33 autosomal linkage groups and chromosome Z with a total of 33,627 markers assigned to one of these linkage groups^33^. To identify markers in PAR, pairwise linkage scores were calculated between 89 markers in the best-order Z chromosome linkage map and 2,904 markers that were not linked to any of the 33 autosomal linkage groups by using TWOPOINT option in CRI-MAP. These 2,904 markers had both heterozygous and homozygous genotypes in males as well as females without deviating from Hardy–Weinberg Equilibrium. Because of being heterozygous in females, they were not included in the initial Z-linkage analysis^33^. Markers that had pairwise LOD score >3.0 with at least one of the 89 Z-linked markers were used for subsequent BUILD analysis to determine their marker order along with the existing Z-chromosome linkage map. Genotypes have been deposited in the Dryad database ( doi:10.5061/dryad.h68jd).

### Identification of the PAR based on sequence coverage

Raw whole-genome re-sequencing reads, obtained by Illumina HiSeq sequencing as described in ref. 31, from 10 female and 10 male collared flycatchers from the above population were mapped to all scaffolds in the FicAlb1.5 assembly version of the collared flycatcher genome (AGTO00000000.2) with Burrows-Wheeler Aligner (BWA) 0.6.2 (ref. 60) using default settings with a soft-clipping base-quality threshold of 5 to avoid low-quality bases in alignments. Alignment quality was enhanced by local realignment with GATK 2.4.3 (ref. 61). Duplicates were marked at the library level using Picard ( http://picard.sourceforge.net).

Base coverage for all Z-linked scaffolds including the three PAR scaffolds (NW_004775940.1 (scaffold N00298), NW_004775959.1 (N00378) and NW_004778032.1 (N02597)) was extracted with SAMtools mpileup 0.1.19 (ref. 62) pooling all individuals from each sex. The scaffolds were divided into 200 kb windows, and the mean and median coverage per window as well as the male-to-female coverage ratio were calculated with in-house scripts. To account for differences in total sequenced reads per sex, we normalized ratios by dividing them with the average M/F ratio of autosomal scaffolds.

### Characterization of the PAR

Gene information was obtained from Ensembl annotation of the FicAlb_1.4 version of the flycatcher genome assembly. The three PAR scaffolds identified in this study correspond to scaffolds JH603441.1 (N00298), JH603380.1 (N00378) and AGTO1003702.1 (N02597) in FicAlb_1.4 available at http://www.ensembl.org. The genome was repeat masked with RepeatMasker (version open-3.2.9) and repeat content and GC content were calculated in 20 kb (for Fig. 1a) or 630 kb windows (=the size of PAR, with 5 kb added to each of the two gaps between the scaffolds, for statistical analysis). Gene expression data for PAR genes was taken from ref. 52 and included expression levels measured by RNA-sequencing in five birds of each sex in brain, kidney, liver, lung, muscle, ovary, skin, testis and embryo (ERX144565-577, ERX144581–585, ERX144589–598, ERX144609–618, ERX144637–650, ERX144661–674, ERX144685–696, ERX144721, ERX144725, ERX144729 and ERX144731). Transcriptome reads were mapped onto the assembly version FicAlb1.5 using TopHat (version 2.0.10) and Cufflinks (version 2.1.1)^63^,^64^.

PAR scaffolds were aligned pairwise to the genomes of chicken (Galgal4) and *Anolis* lizard (AnoCar2.0) using LASTZ^65^. Homologous regions were identified, extracted and ordered to minimize the number of inversions between species. All anchors between each species pair falling in the extracted regions were plotted with R.

### Molecular evolutionary and population genomic analysis

We identified and downloaded putatively orthologous genes from collared flycatcher, zebra finch and chicken through the Biomart ( http://www.biomart.org) retrieval tool in Ensembl release 73 ( http://www.ensembl.org). Codon-based alignments were made using PRANK (v.130410)^66^ with a free-ratio-model in the codeml program in the Phylogenetic Analysis by Maximum Likelihood (PAML4.7) package^67^ to estimate flycatcher lineage-specific *d*_S_ and *d*_N_/*d*_S_ for each gene.

Differentiation (*F*_ST_) between species (using whole-genome re-sequencing data from 10 males and 10 females of the closely related species pied flycatcher) or sexes was estimated using the hierfstat package in R^68^. The proportion of fixed differences between species (*d*_f_) and genetic diversity within species (*π*) were estimated using custom R scripts. Genotypes were assumed to be diploid for the PAR, and haploid for the remainder of the Z chromosome. These parameters were estimated for 20 or 630 kb windows. To investigate the pattern of LD, we first reconstructed haplotypes by Beagle 4 (ref. 69) with 40 iterations for estimating genotype phase, 10 iterations for imputing missing genotypes and 20 haplotype sampling during each iteration. Pairwise LD (*r*^2^) was then calculated for all pairs of SNPs within 20 kb using VCFTools 0.1.12 (ref. 70), and the level of LD within 20 kb windows was estimated by *E*(*r*^2^)=1/(1+*αd*), where *α* is a LD decay parameter over distance *d* between markers.

## Author contributions

L.S. performed read coverage and other bioinformatic analyses, T.K. performed linkage analysis, R.B. performed read mapping and population genetic analysis, P.B. performed molecular evolutionary analysis and S.U. performed gene expression analysis. A.H. provided samples and A.Q. organized long-term flycatcher field studies. H.E. conceived of and led the study, and wrote the manuscript with input from the other authors.

## Additional information

**Accession Codes.** Re-sequencing data have been deposited in the European Nucleotide Archive at EMBL-EBI under the accession codes ERR637360 to ERR637378 and ERR637485 to ERR63752; project code PRJEB7359.

**How to cite this article:** Smeds, L. *et al.* Genomic identification and characterization of the pseudoautosomal region in highly differentiated avian sex chromosomes. *Nat. Commun.* 5:5448 doi: 10.1038/ncomms6448 (2014).

## Supplementary Material

Supplementary Information Supplementary Figures 1-3, Supplementary Tables 1-2, Supplementary Notes 1-3 and Supplementary References

## Figures and Tables

**Table 1:** Genetic map of the PAR and neighbouring region of the Z chromosome.

**Marker (scaffold:position)**	**Cumulative map position (cM)**		
	**Sex average**	**Female**	**Male**
N00298:53720	0	0	0
N00298:169577	0	0	0
N00298:235485	1.3	6.6	0
N00378:45469	4.4	6.6	1.5
N00378:115359	7.9	14.1	1.5
N00378:154206	17.1	44.9	2.6
N02597:626	31.1	64.3	7.3
N00781:8697	31.1	64.3	7.3
N00497:36583	31.6	64.3	9.5
N00058:5243596	32.4	64.3	11.4
N00058:5170038	33.0	64.3	12.8
N00058:5137590	33.3	64.3	13.6
N00058:5074792	33.7	64.3	14.4
N00058:4983582	34.5	64.3	16.0
N00058:4856163	34.8	64.3	16.6
N00058:4693005	35.0	64.3	17.2

The best-order genetic map for the distal part (1.5 Mb) of the collared flycatcher Z chromosome with cumulative linkage position in sex-averaged and sex-specific maps. The pseudoautosomal region (PAR) is represented by scaffolds N00298, N00378 and N02597. Scaffold N00058 is 5.3 Mb, of which only markers from the distal 0.5 Mb are shown. Note that female recombination on the Z chromosome is limited to scaffolds N00298, N00378 and N02597.

**Table 2:** Genomic parameters of the flycatcher PAR.

**Parameter**	**PAR**	**Z chromosome**	**Autosomes**
Female recombination rate (cM/Mb)	102.1	—	3.0
Male recombination rate (cM/Mb)	11.6	2.8	3.3
GC content (%)	49.2	39.5	41.0
Repeat content (per bp)	0.063	0.113	0.121
Expression breadth (*τ*)	0.601	0.657	0.645
Male:female expression ratio	0.95	1.40	1.02
Synonymous substitution rate (*d*_S_)	0.13	0.78	0.090
*d*_N_/*d*_S_	0.095	0.170	0.169
Nucleotide diversity (*π*)	0.0034	0.0032	0.0037
Collared flycatcher-pied flycatcher *F*_ST_	0.372	0.555	0.357
Male–female collared flycatcher *F*_ST_	0.007	0.012	ND

PAR, pseudoautosomal region.

The values provided are mean values.

## References

[b1] Bachtrog D. Y-chromosome evolution: emerging insights into processes of Y-chromosome degeneration. Nat. Rev. Genet. 14, 113–124 (2013). 23329112 10.1038/nrg3366 PMC4120474

[b2] Charlesworth B. & Charlesworth D. The degeneration of Y chromosomes. Phil. Trans. R. Soc. Lond. Ser. B Biol. Sci. 355, 1563–1572 (2000). 11127901 10.1098/rstb.2000.0717 PMC1692900

[b3] Mank J. E. Small but mighty: the evolutionary dynamics of W and Y sex chromosomes. Chromosome Res. 20, 21–33 (2012). 22038285 10.1007/s10577-011-9251-2 PMC3299550

[b4] Rice W. R. Genetic hitchhiking and the evolution of reduced genetic activity of the Y sex chromosome. Genetics 116, 161–167 (1987). 3596229 10.1093/genetics/116.1.161 PMC1203114

[b5] Rice W. R. Degeneration of a nonrecombining chromosome. Science 263, 230–232 (1994). 8284674 10.1126/science.8284674

[b6] Bachtrog D. The temporal dynamics of processes underlying Y chromosome degeneration. Genetics 179, 1513–1525 (2008). 18562655 10.1534/genetics.107.084012 PMC2475751

[b7] Mohandas T. *et al.* Role of the pseudoautosomal region in sex-chromosome pairing during male meiosis: meiotic studies in a man with a deletion of distal Xp. Am. J. Hum. Genet. 51, 526–533 (1992). 1496984 PMC1682713

[b8] Gabriel-Robez O. *et al.* Deletion of the pseudoautosomal region and lack of sex-chromosome pairing at pachytene in two infertile men carrying an X;Y translocation. Cytogenet. Cell Genet. 54, 38–42 (1990). 2249473 10.1159/000132951

[b9] Otto S. P. *et al.* About PAR: the distinct evolutionary dynamics of the pseudoautosomal region. Trends Genet. 27, 358–367 (2011). 21962971 10.1016/j.tig.2011.05.001

[b10] Graves J. A. M. Mammals that break the rules: genetics of marsupials and monotremes. Annu. Rev. Genet. 30, 233–260 (1996). 8982455 10.1146/annurev.genet.30.1.233

[b11] Handel M. A. The XY body: a specialized meiotic chromatin domain. Exp. Cell Res. 296, 57–63 (2004). 15120994 10.1016/j.yexcr.2004.03.008

[b12] Checchi P. M. & Engebrecht J. Heteromorphic sex chromosomes: navigating meiosis without a homologous partner. Mol. Reprod. Dev. 78, 623–632 (2011). 22113949 10.1002/mrd.21369 PMC3223601

[b13] Koller P. & Darlington C. The genetical and mechanical properties of the sex chromosomes. I *Rattus norvegicus*. J. Genet. 29, 159–173 (1934).

[b14] Bergero R., Forrest A., Kamau E. & Charlesworth D. Evolutionary strata on the X chromosomes of the dioecious plant *Silene latifolia*: evidence from new sex-linked genes. Genetics 175, 1945–1954 (2007). 17287532 10.1534/genetics.106.070110 PMC1855140

[b15] Page D. C., Harper M. E., Love J. & Botstein D. Occurrence of a transposition from the X-chromosome long arm to the Y-chromosome short arm during human evolution. Nature 311, 119–123 (1984). 6088994 10.1038/311119a0

[b16] Raudsepp T., Das P. J., Avila F. & Chowdhary B. P. The pseudoautosomal region and sex chromosome aneuploidies in domestic species. Sex. Dev. 6, 72–83 (2012). 21876343 10.1159/000330627

[b17] Flaquer A., Rappold G. A., Wienker T. F. & Fischer C. The human pseudoautosomal regions: a review for genetic epidemiologists. Eur. J. Hum. Genet. 16, 771–779 (2008). 18398439 10.1038/ejhg.2008.63

[b18] Ellegren H. Evolution of the avian sex chromosomes and their role in sex determination. Trends Ecol. Evol. 15, 188–192 (2000). 10782132 10.1016/s0169-5347(00)01821-8

[b19] Ellegren H. Sex-chromosome evolution: recent progress and the influence of male and female heterogamety. Nat. Rev. Genet. 12, 157–166 (2011). 21301475 10.1038/nrg2948

[b20] ICGSC. Sequence and comparative analysis of the chicken genome provide unique perspectives on vertebrate evolution. Nature 432, 695–716 (2004). 15592404 10.1038/nature03154

[b21] Ellegren H. & Carmichael A. Multiple and independent cessation of recombination between avian sex chromosomes. Genetics 158, 325–331 (2001). 11333240 10.1093/genetics/158.1.325 PMC1461649

[b22] Lawson Handley L., Ceplitis H. & Ellegren H. Evolutionary strata on the chicken Z chromosome: implications for sex chromosome evolution. Genetics 167, 367–376 (2004). 15166161 10.1534/genetics.167.1.367 PMC1470863

[b23] Nam K. & Ellegren H. The chicken (*Gallus gallus*) Z chromosome contains at least three nonlinear evolutionary strata. Genetics 180, 1131–1136 (2008). 18791248 10.1534/genetics.108.090324 PMC2567362

[b24] Wright A. E., Moghadam H. K. & Mank J. E. Trade-off between selection for dosage compensation and masculinization on the avian Z chromosome. Genetics 192, 1433–1445 (2012). 22997237 10.1534/genetics.112.145102 PMC3512148

[b25] Warren W. C. *et al.* The genome of a songbird. Nature 464, 757–762 (2010). 20360741 10.1038/nature08819 PMC3187626

[b26] Vicoso B., Kaiser V. B. & Bachtrog D. Sex-biased gene expression at homomorphic sex chromosomes in emus and its implication for sex chromosome evolution. Proc. Natl Acad. Sci. USA 110, 6453–6458 (2013). 23547111 10.1073/pnas.1217027110 PMC3631621

[b27] Papoli H. Y. & Ellegren H. Old but not (so) degenerated: slow evolution of largely homomorphic sex chromosomes in ratites. Mol. Biol. Evol. 31, 1444–1453 (2014). 24618361 10.1093/molbev/msu101

[b28] Kirkpatrick M. & Guerrero R. F. Signatures of sex-antagonistic selection on recombining sex chromosomes. Genetics 197, 531–541 (2014). 24578352 10.1534/genetics.113.156026 PMC4063913

[b29] Charlesworth B., Jordan C. & Charlesworth D. The evolutionary dynamics of sexually antagonistic mutations in pseudoautosomal regions of sex chromosomes. Evolution 68, 1339–1350 (2014). 24476564 10.1111/evo.12364 PMC4289941

[b30] Kirkpatrick M., Guerrero R. F. & Scarpino S. V. Patterns of neutral genetic variation on recombining sex chromosomes. Genetics 184, 1141–1152 (2010). 20124026 10.1534/genetics.109.113555 PMC2865914

[b31] Ellegren H. *et al.* The genomic landscape of species divergence in Ficedula flycatchers. Nature 491, 756–760 (2012). 23103876 10.1038/nature11584

[b32] Kawakami T. *et al.* Estimation of linkage disequilibrium and interspecific gene flow in *Ficedula* flycatchers by a newly developed 50k SNP array. Mol. Ecol. Res. 14, 4035–4058 (2014). 10.1111/1755-0998.12270 PMC4368375 24784959

[b33] Kawakami T. *et al.* A high-density linkage map enables a second-generation collared flycatcher genome assembly and reveals the patterns of avian recombination rate variation and chromosomal evolution. Mol. Ecol. 23, 4035–4058 (2014). 24863701 10.1111/mec.12810 PMC4149781

[b34] Roesti M., Moser D. & Berner D. Recombination in the threespine stickleback genome—patterns and consequences. Mol. Ecol. 22, 3014–3027 (2013). 23601112 10.1111/mec.12322

[b35] Vicoso B., Emerson J. J., Zektser Y., Mahajan S. & Bachtrog D. Comparative sex chromosome genomics in snakes: differentiation, evolutionary strata, and lack of global dosage compensation. PLoS Biol. 11, e1001643 (2013). 24015111 10.1371/journal.pbio.1001643 PMC3754893

[b36] Chen S. *et al.* Whole-genome sequence of a flatfish provides insights into ZW sex chromosome evolution and adaptation to a benthic lifestyle. Nat. Genet. 46, 253–260 (2014). 24487278 10.1038/ng.2890

[b37] Nam K. & Ellegren H. Recombination drives vertebrate genome contraction. PLoS Genet. 8, e1002680 (2012). 22570634 10.1371/journal.pgen.1002680 PMC3342960

[b38] Montoya-Burgos J. I., Boursot P. & Galtier N. Recombination explains isochores in mammalian genomes. Trends Genet. 19, 128–130 (2003). 12615004 10.1016/S0168-9525(03)00021-0

[b39] Duret L. & Arndt P. F. The impact of recombination on nucleotide substitutions in the human genome. PLoS Genet. 4, e1000071 (2008). 18464896 10.1371/journal.pgen.1000071 PMC2346554

[b40] Fullerton S. M., Bernardo Carvalho A. & Clark A. G. Local rates of recombination are positively correlated with GC content in the human genome. Mol. Biol. Evol. 18, 1139–1142 (2001). 11371603 10.1093/oxfordjournals.molbev.a003886

[b41] Mugal C. F., Arndt P. F. & Ellegren H. Twisted signatures of GC-biased gene conversion embedded in an evolutionary stable karyotype. Mol. Biol. Evol. 30, 1700–1712 (2013). 23564940 10.1093/molbev/mst067 PMC3684855

[b42] Künstner A., Nabholz B. & Ellegren H. Significant selective constraint at 4-fold degenerate sites in the avian genome and its consequence for detection of positive selection. Genome Biol. Evol. 3, 1381–1389 (2011). 22042333 10.1093/gbe/evr112 PMC3242499

[b43] Ellegren H. Characteristics, causes and evolutionary consequences of male-biased mutation. Proc. R. Soc. Ser. B Biol. Sci. 274, 1–10 (2007). 10.1098/rspb.2006.3720 PMC1679872 17134994

[b44] Schoenmakers S. *et al.* Female meiotic sex chromosome inactivation in chicken. PLoS Genet. 5, e1000466 (2009). 19461881 10.1371/journal.pgen.1000466 PMC2678266

[b45] Guioli S., Lovell-Badge R. & Turner J. M. A. Error-prone ZW pairing and no evidence for meiotic sex chromosome inactivation in the chicken germ line. PLoS Genet. 8, e1002560 (2012). 22412389 10.1371/journal.pgen.1002560 PMC3297585

[b46] Arnqvist G. & Rowe L. Sexual Conflict Princeton Univ. Press (2005).

[b47] Jordan C. & Charlesworth D. The potential for sexually antagonistic polymorphism in different genome regions. Evolution 66, 505–516 (2012). 22276544 10.1111/j.1558-5646.2011.01448.x

[b48] Qiu S., Bergero R. & Charlesworth D. Testing for the footprint of sexually antagonistic polymorphisms in the pseudoautosomal region of a plant sex chromosome pair. Genetics 194, 663–672 (2013). 23733787 10.1534/genetics.113.152397 PMC3697971

[b49] Martin S. H. *et al.* Genome-wide evidence for speciation with gene flow in Heliconius butterflies. Genome Res. 23, 1817–1828 (2013). 24045163 10.1101/gr.159426.113 PMC3814882

[b50] Rice W. R. Sex-chromosomes and evolution of sexualdimorphism. Evolution 38, 735–742 (1984). 10.1111/j.1558-5646.1984.tb00346.x 28555827

[b51] Griffin R. M., Dean R., Grace J. L., Rydén P. & Friberg U. The shared genome is a pervasive constraint on the evolution of sex-biased gene expression. Mol. Biol. Evol. 30, 2168–2176 (2013). 23813981 10.1093/molbev/mst121

[b52] Uebbing S., Künstner A., Mäkinen H. & Ellegren H. Transcriptome sequencing reveals the character of incomplete dosage compensation across multiple tissues in flycatchers. Genome Biol. Evol. 5, 1555–1566 (2013). 23925789 10.1093/gbe/evt114 PMC3762201

[b53] Itoh Y. *et al.* Dosage compensation is less effective in birds than in mammals. J. Biol. 6, 2 (2007). 17352797 10.1186/jbiol53 PMC2373894

[b54] Ellegren H. *et al.* Faced with inequality: chicken do not have a general dosage compensation of sex-linked genes. BMC Biol. 5, 40 (2007). 17883843 10.1186/1741-7007-5-40 PMC2099419

[b55] Nabholz B., Kunstner A., Wang R., Jarvis E. & Ellegren H. Dynamic evolution of base composition: causes and consequences in avian phylogenomics. Mol. Biol. Evol. 28, 2197–2210 (2011). 21393604 10.1093/molbev/msr047 PMC3144382

[b56] Ellegren H. The evolutionary genomics of birds. Annu. Rev. Ecol. Evol. Syst. 44, 239–259 (2013).

[b57] Winckler W. *et al.* Comparison of fine-scale recombination rates in humans and chimpanzees. Science 308, 107–111 (2005). 15705809 10.1126/science.1105322

[b58] Charlesworth D., Charlesworth B. & Marais G. Steps in the evolution of heteromorphic sex chromosomes. Heredity 95, 118–128 (2005). 15931241 10.1038/sj.hdy.6800697

[b59] Green P., Falls K. & Crook S. Documentation for CRIMAP, version 2.4 Washington Univ. School of Medicine (1990).

[b60] Li H. & Durbin R. Fast and accurate long-read alignment with Burrows-Wheeler transform. Bioinformatics 26, 589–595 (2010). 20080505 10.1093/bioinformatics/btp698 PMC2828108

[b61] McKenna A. *et al.* The Genome Analysis Toolkit: a MapReduce framework for analyzing next-generation DNA sequencing data. Genome Res. 20, 1297–1303 (2010). 20644199 10.1101/gr.107524.110 PMC2928508

[b62] Li H. *et al.* The Sequence Alignment/Map format and SAMtools. Bioinformatics 25, 2078–2079 (2009). 19505943 10.1093/bioinformatics/btp352 PMC2723002

[b63] Kim D. *et al.* TopHat2: accurate alignment of transcriptomes in the presence of insertions, deletions and gene fusions. Genome Biol. 14, R36 (2013). 23618408 10.1186/gb-2013-14-4-r36 PMC4053844

[b64] Trapnell C. *et al.* Differential gene and transcript expression analysis of RNA-seq experiments with TopHat and Cufflinks. Nat. Protoc. 7, 562–578 (2012). 22383036 10.1038/nprot.2012.016 PMC3334321

[b65] Harris R. S. Improved pairwise alignment of genomic DNA PhD thesis, Pennsylvania State Univ. (2007).

[b66] Löytynoja A. & Goldman N. An algorithm for progressive multiple alignment of sequences with insertions. Proc. Natl Acad. Sci. USA 102, 10557–10562 (2005). 16000407 10.1073/pnas.0409137102 PMC1180752

[b67] Yang Z. PAML 4: phylogenetic analysis by maximum likelihood. Mol. Biol. Evol. 24, 1586–1591 (2007). 17483113 10.1093/molbev/msm088

[b68] Goudet J. hierfstat, a package for r to compute and test hierarchical F-statistics. Mol. Ecol. Notes 5, 184–186 (2005).

[b69] Browning B. L. & Browning S. R. Improving the accuracy and efficiency of identity-by-descent detection in population data. Genetics 194, 459–471 (2013). 23535385 10.1534/genetics.113.150029 PMC3664855

[b70] Danecek P. *et al.* The variant call format and VCFtools. Bioinformatics 27, 2156–2158 (2011). 21653522 10.1093/bioinformatics/btr330 PMC3137218

