# Supplementary material for: Genomic identification and characterization of the pseudoautosomal region in highly differentiated avian sex chromosomes
Source: Nat Commun. 2014 Nov 7;5:5448. doi: 10.1038/ncomms6448 (PMC4272252; doi:10.1038/ncomms6448)
Supplement: Supplementary Information — Supplementary Figures 1-3, Supplementary Tables 1-2, Supplementary Notes 1-3 and Supplementary References [file ncomms6448-s1.pdf]

A) Female

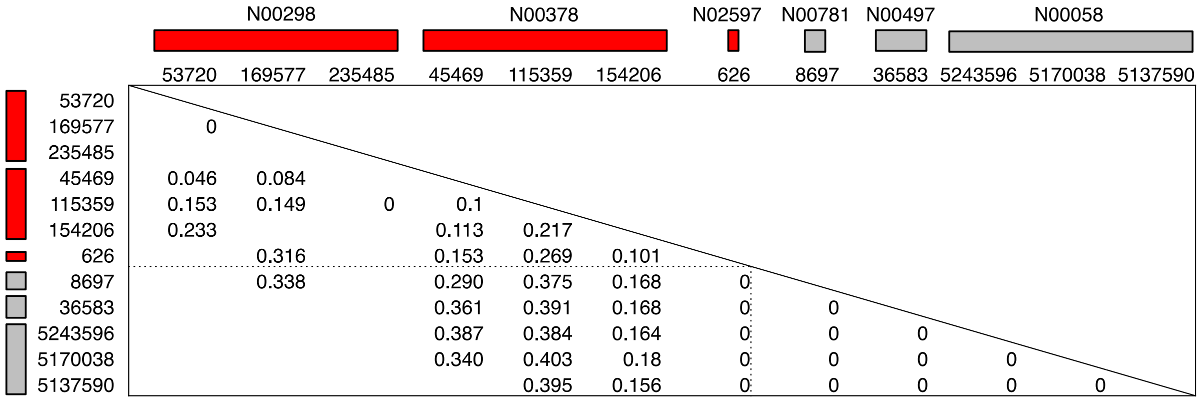

B) Male

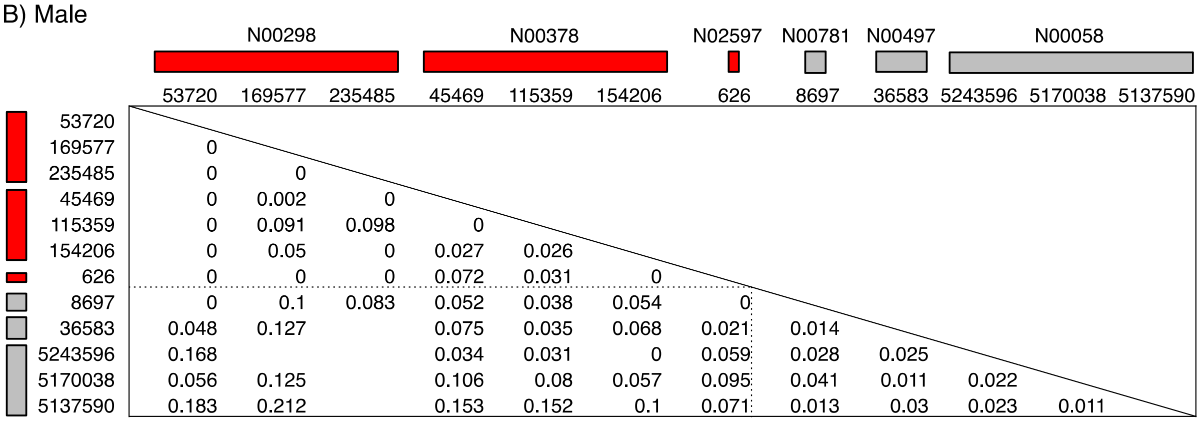

Supplementary Figure 1. Pairwise recombination fractions between SNP markers in the pseudoautosomal region (PAR) and neighbouring SNP markers on sex-specific part of Z chromosome for A) females and B) males. Red and grey rectangles indicate PAR and Z chromosome scaffolds, respectively. Numbers below scaffolds indicate SNP positions on scaffolds. The border between PAR and Z chromosome is indicated by dotted line. Only recombination fractions with LOD score support larger than 3 are shown.

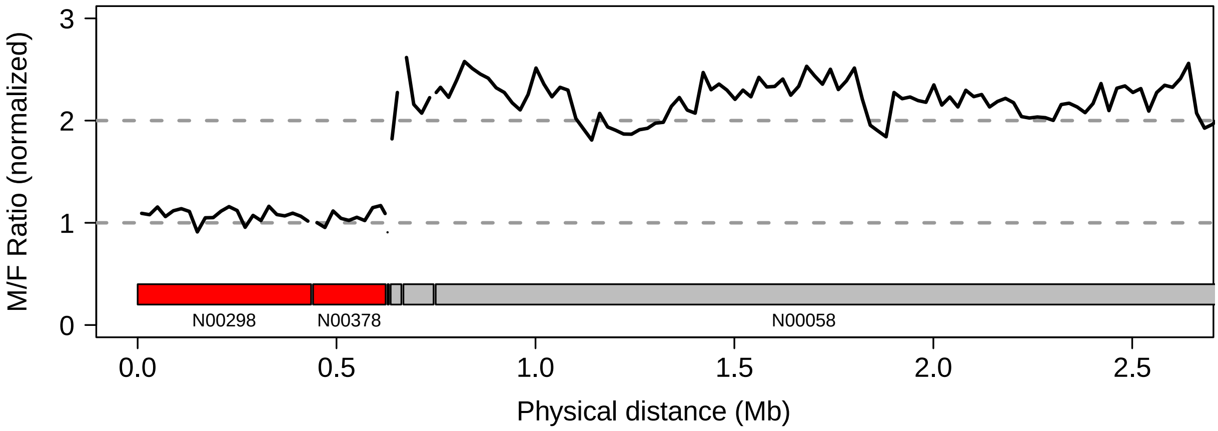

Supplementary Figure 2. Male-to-female (M/F) coverage ratio for 20 kb windows in the distal part of the Z chromosome. Scaffolds are indicated below the plot. Coverage was normalized by the average M/F ratio of autosomal scaffolds.

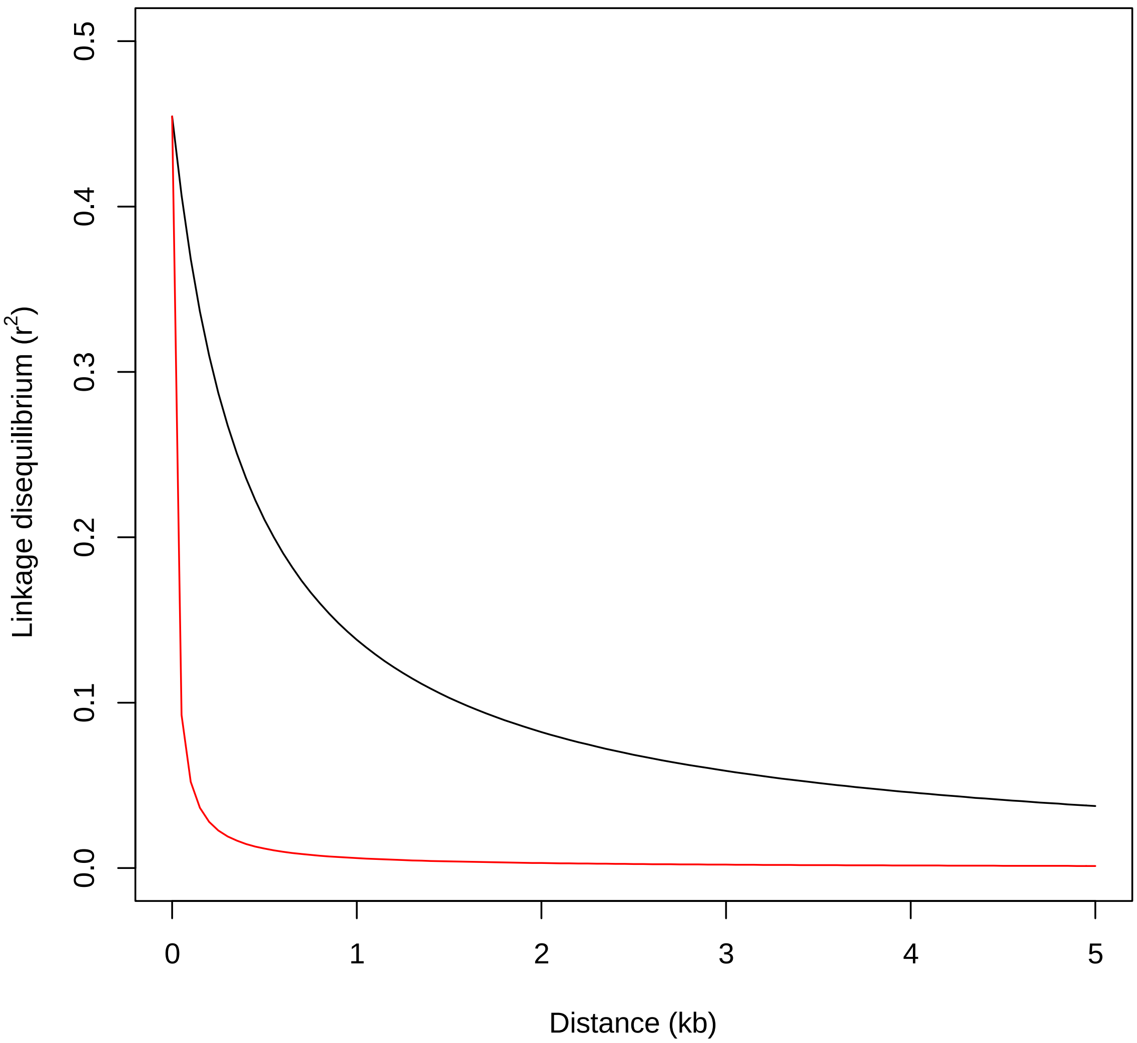

Supplementary Figure 3. Relationship between level of linkage disequilibrium (r^2) and physical distance between pairs of markers (LD decay curve). Red is the PAR and black is the rest of the Z chromosome. The LD decay curve was estimated by nonlinear regression[1].

Supplementary Table 1. Significant two-point linkage between markers from the PAR and markers on the Z chromosome. For each marker from the PAR, linkage to the Z chromosome marker with the strongest LOD score support from three different scaffolds is listed.

PAR marker	Linked Z marker	Female		Male		Sex-average	
(scaffold:position)		Rec	LOD	Rec	LOD	Rec	LOD
N00298:53720	N00781:8697	0.483	0.89	0	19.27	0.259	2.79
	N00497:36583	0.500	0	0.048	23.51	0.265	3.55
	N00058:5170038	0.500	0	0.056	20.33	0.279	2.84
N00298:169577	N00781:8697	0.338	3.28	0.1	4.73	0.258	1.57
	N00497:36583	0.500	0	0.127	4.88	0.273	1.75
	N00058:5170038	0.500	0	0.125	5.24	0.293	1.09
N00298:235485	N00781:8697	0.497	0.01	0.083	4.76	0.224	1.44
	N00497:36583	0.470	0.11	0.256	0.80	0.457	0.02
	N00058:5170038	0.459	0.22	0.229	2.48	0.313	0.60
N00378:45469	N00781:8697	0.290	8.58	0.052	22.9	0.154	6.70
	N00497:36583	0.361	6.89	0.075	18.08	0.187	5.39
	N00058:5170038	0.340	6.49	0.106	21.03	0.195	5.36
N00378:115359	N00781:8697	0.375	4.60	0.038	20.78	0.210	4.79
	N00497:36583	0.391	4.24	0.035	21.97	0.205	5.66
	N00058:5170038	0.403	3.47	0.080	20.65	0.227	4.51
N00378:154206	N00781:8697	0.168	11.35	0.054	12.61	0.117	10.89
	N00497:36583	0.168	11.25	0.068	14.93	0.123	11.91
	N00058:5170038	0.180	10.75	0.057	14.26	0.126	11.61
N02597:626	N00781:8697	0	27.09	0	17.16	0	26.49

	N00497:36583	0	30.10	0.021	13.50	0.008	24.24
	N00058:5170038	0	29.80	0.095	11.39	0.042	21.08

Supplementary Table 2. List of genes in the collared flycatcher PAR, their position in the Z chromosome assembly and male-to-female (M:F) expression ratio.

Gene symbol	Gene	Ensembl ID	Starts (bp)	Ends (bp)	M:F ratio
Novel		ENSFALG00000011552	7885	3068	0.76
Novel		ENSFALG00000011557	61860	15716	0.94
Novel		ENSFALG00000011561	138014	137043	0.81
TXNL1	thioredoxin-like 1	ENSFALG00000011580	163014	148969	0.97
WDR7	WD repeat domain 7	ENSFALG00000011623	168591	202971	0.91
ST8SIA3	ST8 alpha-N-acetyl-neuraminide alpha-2,8-sialyltransferase 3	ENSFALG00000011668	235245	238102	1.20
Novel		ENSFALG00000011567	243179	147380	-
ONECUT2	one cut homeobox 2	ENSFALG00000011678	247536	257035	0.76
Novel		ENSFALG00000011685	267155	262688	0.92
FECH	ferrochelatase	ENSFALG00000011690	292084	284455	0.91
NARS	asparaginyl-tRNA synthetase	ENSFALG00000011698	303598	298472	1.01
ATP8B1	ATPase, aminophospholipid transporter, class I, type 8B, member 1	ENSFALG00000011710	320466	306830	1.04
NEDD4L	neural precursor cell expressed, developmentally down-regulated 4-like	ENSFALG00000011721	362872	427848	1.07
LMAN1	lectin, mannose-binding, 1	ENSFALG00000009746	458778	471081	1.04

CPLX4	complexin 4	ENSFALG00000009751	475285	483012	1.03
RAX	retina and anterior neural fold homeobox	ENSFALG00000009755	491593	493031	0.82
GRP	gastrin-releasing peptide	ENSFALG00000009759	503864	499306	1.07
SEC11C	SEC11 homolog C	ENSFALG00000009763	511985	508340	0.95
ZNF532	zinc finger protein 532	ENSFALG00000009766	541001	517686	0.99
MALT1	mucosa associated lymphoid tissue lymphoma translocation gene 1	ENSFALG00000009773	578833	562895	0.97
ALPK2	alpha-kinase 2	ENSFALG00000009783	603034	608770	0.88
Novel		ENSFALG00000015559	610043	609952	-

Supplementary Note 1. Sex chromosomes in paleognath birds.

The deepest split within modern birds is that between Palaeognathae and Neognathae, with the latter containing >99% of all extant avian species. Cytogenetic work has shown that recombination in female meiosis occurs between large parts of the Z chromosome and the W chromosome in ratites [2-4], the group of flightless birds within Palaeognathae that includes ostrich, kiwi, emu and others. A recent RNA-seq study estimated that approximately two-thirds of the ostrich Z chromosome recombines in female meiosis [5] and this might also be the case in emus [6]. In these species the PAR thus constitute the majority of the Z chromosome (cf. [7]. Since the sex chromosomes in Palaeognathae and Neognathae seem fully homologous [8,9] and because Z-W differentiation started prior to the split of the two lineages 130-150 million years ago [5,10,11], sex chromosome evolution in Palaeognathae has been slow. In contrast, sex chromosome evolution in Neognathae has resulted in advanced Z-W differentiation. This difference may be related to an absence of dosage compensation in non-recombining regions of the ostrich Z chromosome, where male expression is on average twofold higher than female expression [5]. Without a compensatory mechanism, there might be strong selection against expansion of the non-recombining region because hemizygosity of sex-linked genes in the heterogametic sex should perturb gene interactions and networks. In non-recombining regions of neognath Z chromosomes, the degree of dosage compensation varies and may have evolved on a gene-by-gene basis, allowing recombination restriction to spread [12-15].

Supplementary Note 2. The flycatcher pseudoautosomal region.

Using the BUILD option for multipoint linkage analysis in CRIMAP, scaffold order N00298-N00378-"all scaffolds from the majority of the Z chromosomes that do not recombine in female meiosis" was supported by a LOD score >5. The order N00298-N00387-N02597-"Z chromosome" was supported by a LOD score >2. The LOD score support for orientation of

N00378 was >3 while for N00298 it was only >0.1. However, independent support for the inferred orientation of N00298 was provided by analyses of mapped mate-pairs from three libraries with insert sizes of 5 kb, 18 kb and 21 kb, respectively (SRA Accession numbers ERX093495, ERX093467 and ERX093470) [16]. Any read that mapped closer to an end than each library's insert size was considered and links between mates on different scaffolds were counted. Mate-pair links were only found between the end of N00298 and the start of N00378. We note that if the orientation of N00298 would be the opposite, it would not affect our recombination rate estimates much since most PAR recombination in females occurred in N00387.

Supplementary Note 3. Evolution of the avian pseudoautosomal region.

Failure to identify the PAR in chicken [17] and zebra finch [18] genome sequencing projects and cytogenetic evidence for recombination being restricted to the terminus of a small Z-W pairing region in birds from other neognath lineages [19], suggests that a small PAR as identified in flycatcher may be a common feature of neognath sex chromosomes. However, since there are deep, multiple splits within Neognathae (which includes some 20 avian orders), in particular in the group of Neoaves, and because it is well known that sex chromosome evolution has occurred in parallel in different neognath lineages [20,21], it cannot be excluded that sex chromosomes of species from avian lineages yet to be investigated are only intermediately differentiated. It should be straightforward to test this by comparing male and female coverage over the Z chromosome in deep genome sequencing, analogous to what we demonstrate in Fig. 2. Because the end of the flycatcher Z chromosome harbouring the PAR corresponds to one of the ends of the distantly related chicken Z chromosome (**Fig. 3**), it is a strong candidate region for representing the PAR across different bird lineages.

In the absence of an assembled flycatcher W chromosome we cannot date the events leading to recombination restriction between sex chromosomes in this lineage. However, the fact that the male-to-female ratio in read coverage was consistently close to two outside the PAR (**Fig. 2**) indicates that, overall, the degree of any remaining Z-W sequence similarity in the region that most recently ceased to recombine was not sufficient for reads from the W chromosome to map to the Z chromosome. Thus, the situation with a very small PAR may have persisted for a long period of time in the lineage leading to flycatcher.

Supplementary References

1. Hill, W.G. & Weir, B.S. Variances and covariances of squared linkage disequilibria in finite populations. *Theoretical Population Biology* **33**, 54-78 (1988).
2. Pigozzi, M.I. & Solari, A.J. Extreme axial equalization and wide distribution of recombination nodules in the primitive ZW pair of Rhea americana (Aves, Ratitae). *Chromosome Research* **5**, 421-428 (1997).
3. Pigozzi, M.I. & Solari, A.J. The ZW pairs of two paleognath birds from two orders show transitional stages of sex chromosome differentiation. *Chromosome Research* **7**, 541-551 (1999).
4. Ogawa, A., Murata, K. & Mizuno, S. The location of Z- and W-linked marker genes and sequence on the homomorphic sex chromosomes of the ostrich and the emu. *Proceedings of the National Academy of Sciences of the United States of America* **95**, 4415-4418 (1998).
5. Papoli, H.Y. & Ellegren, H. Old but not (so) degenerated: slow evolution of largely homomorphic sex chromosomes in ratites. *Molecular Biology and Evolution* **31**, 1444-1453 (2014).
6. Vicoso, B., Kaiser, V.B. & Bachtrog, D. Sex-biased gene expression at homomorphic sex chromosomes in emus and its implication for sex chromosome evolution.

Proceedings of the National Academy of Sciences of the United States of America **110**, 6453-6458 (2013).

7. Janes, D.E., Ezaz, T., Graves, J.A.M. & Edwards, S.V. Recombination and nucleotide diversity in the sex chromosomal pseudoautosomal region of the emu, *Dromaius novaehollandiae*. *Journal of Heredity* **100**, 125-136 (2009).
8. Nishida-Umehara, C. *et al.* The molecular basis of chromosome orthologies and sex chromosomal differentiation in palaeognathous birds. **15**, 721-734 (2007).
9. Shetty, S., Griffin, D.K. & Graves, J.A.M. Comparative painting reveals strong chromosome homology over 80 million years of bird evolution. *Chromosome Research* **7**, 289-295 (1999).
10. Wright, A.E., Moghadam, H.K. & Mank, J.E. Trade-off between selection for dosage compensation and masculinization on the avian Z chromosome. *Genetics* **192**, 1433-1445 (2012).
11. Nam, K. & Ellegren, H. The chicken (*Gallus gallus*) Z chromosome contains at least three nonlinear evolutionary strata. *Genetics* **180**, 1131-1136 (2008).
12. Mank, J.E. & Ellegren, H. All dosage compensation is local: Gene-by-gene regulation of sex-biased expression on the chicken Z chromosome. *Heredity* **102**, 312-320 (2009).
13. Wolf, J. & Bryk, J. General lack of global dosage compensation in ZZ/ZW systems? Broadening the perspective with RNA-seq. *BMC Genomics* **12**, 91 (2011).
14. Uebbing, S., Künstner, A., Mäkinen, H. & Ellegren, H. Transcriptome sequencing reveals the character of incomplete dosage compensation across multiple tissues in flycatchers. *Genome Biology and Evolution* **5**, 1555-1566 (2013).
15. Naurin, S., Hasselquist, D., Bensch, S. & Hansson, B. Sex-biased gene expression on the avian Z chromosome: Highly expressed genes show higher male-biased expression. *PLoS ONE* **7**, e46854 (2012).
16. Ellegren, H. *et al.* The genomic landscape of species divergence in Ficedula flycatchers. *Nature* **491**, 756-760 (2012).
17. ICGSC. Sequence and comparative analysis of the chicken genome provide unique perspectives on vertebrate evolution. *Nature* **432**, 695-716 (2004).
18. Warren, W.C. *et al.* The genome of a songbird. *Nature* **464**, 757-762 (2010).
19. Solari, A.J. & Pigozzi, M.I. Recombination nodules and axial equalization in the ZW pairs of the Peking duck and the Guinea fowl. *Cytogenetic and Genome Research* **64**, 268-272 (1993).

20. de Kloet, R.S. & de Kloet, S.R. Evolution of the spindlin gene in birds: Independent cessation of the recombination of sex chromosomes at the spindlin locus in Neognathous birds and Tinamous, a Palaeognathous avian family. *Genetica* **119**, 333-342 (2003).
21. Ellegren, H. & Carmichael, A. Multiple and independent cessation of recombination between avian sex chromosomes. *Genetics* **158**, 325-331 (2001).
